# *In Silico* Sperm Proteome Analysis to Investigate DNA Repair Mechanisms in Varicocele Patients

**DOI:** 10.3389/fendo.2021.757592

**Published:** 2021-12-17

**Authors:** Renata Finelli, Sara Darbandi, Peter Natesan Pushparaj, Ralf Henkel, Edmund Ko, Ashok Agarwal

**Affiliations:** ^1^ American Center for Reproductive Medicine, Cleveland Clinic, Cleveland, OH, United States; ^2^ Fetal Health Research Center, Hope Generation Foundation, Tehran, Iran; ^3^ Gene Therapy and Regenerative Medicine Research Center, Hope Generation Foundation, Tehran, Iran; ^4^ Center of Excellence in Genomic Medicine Research and Department of Medical Laboratory Technology, Faculty of Applied Medical Sciences, King Abdulaziz University, Jeddah, Saudi Arabia; ^5^ Department of Metabolism, Digestion and Reproduction, Imperial College London, London, United Kingdom; ^6^ Department of Medical Bioscience, University of the Western Cape, Bellville, South Africa; ^7^ LogixX Pharma, Reading, United Kingdom; ^8^ Department of Urology, Loma Linda University Health, Loma Linda, CA, United States

**Keywords:** DNA damage, DNA repair, male infertility, proteomics, varicocele

## Abstract

Varicocele, a condition associated with increased oxidative stress, negatively affects sperm DNA integrity and reduces pregnancy rates. However, the molecular mechanisms related to DNA integrity, damage, and repair in varicocele patients remain unclear. This study aimed to determine the role of DNA repair molecular mechanisms in varicocele-related infertility by combining an *in silico* proteomics approach with wet-laboratory techniques. Proteomics results previously generated from varicocele patients (n=50) and fertile controls (n=10) attending our Andrology Center were reanalyzed using bioinformatics tools, including the WEB-based Gene SeT AnaLysis Toolkit, Open Target Platform, and Ingenuity Pathway Analysis (IPA), to identify differentially expressed proteins (DEPs) involved in DNA repair. Subsequently, selected DEPs in spermatozoa were validated using western blotting in varicocele (n = 13) and fertile control (n = 5) samples. We identified 99 DEPs mainly involved in male reproductive system disease (n=66) and male infertility (n=47). IPA analysis identified five proteins [fatty acid synthase (FASN), myeloperoxidase (MPO), mitochondrial aconitate hydratase (ACO2), nucleoporin 93 (NUP93), and 26S proteasome non-ATPase regulatory subunit 14 (PSMD14)] associated with DNA repair deficiency, which showed altered expression in varicocele (P <0.03). We validated ACO2 downregulation (fold change=0.37, change%=-62.7%, P=0.0001) and FASN overexpression (fold change = 4.04, change %= 303.7%, P = 0.014) in men with varicocele compared to controls. This study combined a unique *in silico* approach with an *in vitro* validation of the molecular mechanisms that may be responsible for varicocele-associated infertility. We identified ACO2 and FASN as possible proteins involved in DNA repair, whose altered expression may contribute to DNA damage in varicocele pathophysiology.

## 1 Introduction

Varicocele pathology is characterized by enlargement of the pampiniform plexus, a complex of small spermatic veins that drain venous blood from the testes into the inferior vena cava (right testicle) or the left renal vein (left testicle). Blood stasis in the enlarged veins results in testicular hypoxia, hypotension, and hyperthermia (1, 2), which significantly affect sperm production, an array of sperm functions (such as capacitation, hyperactivation, and acrosome reaction), and consequently fertility potential (3, 4). Increased oxidative stress is considered one of the main contributors to infertility in patients with varicocele (3). This is characterized by high levels of reactive oxygen species (ROS) that can negatively affect sperm functions through the oxidation of lipids, proteins, and DNA, resulting in increased membrane instability, loss of protein function, DNA damage, and ultimately reduced pregnancy rate (5, 6). Although varicocele patients have been reported to have abnormal semen quality, some patients can be normozoospermic (4). Considering the impact of oxidative stress on DNA integrity (7), it is not surprising that several studies reported high levels of sperm DNA damage in varicocele patients, regardless of their fertility status (8). Moreover, varicocele patients showed a significant improvement in sperm DNA fragmentation (SDF) values after varicocele repair (9–12), and consequently improved pregnancy rate (13). Hence, recent guideline has recommended SDF testing in case of clinical varicocele to help the clinician choosing the best therapeutic plan (8).

In spermatozoa, the organization of nuclear chromatin differs from that of somatic cells (14). During spermatogenesis, approximately 85% of the DNA histones are replaced by protamines, P1 and P2. P1 and P2 are basic proteins that condense chromatin up to 20 times more than in somatic cells through their cysteine disulfide bonds and protect DNA from external insults (15). As P1 and P2 are usually present in a ratio between 0.8 to 1.2, any alteration in their levels can increase the DNA susceptibility to damage and result in decreased fertilization and embryo development rates (16–18). Repair of sperm DNA does not occur in mature sperm; in spermatids, DNA damage is repaired by nucleotide excision repair (NER) and base excision repair (BER) mechanisms (19). Furthermore, spermatids can rely on the mismatch repair (MMR) mechanism for removing base-base mismatches and insertions/deletions, while DNA double-strand breaks (DSBs) are repaired through non-homologous end joining (NHEJ) and alternative non-homologous end joining (A-NHEJ) pathways (19).

Considering the adverse impact of DNA damage on male fertility and reproduction (20–23), the investigation of the mechanisms involved in DNA repair in varicocele patients is of interest to elucidate and/or clarify the molecular mechanisms leading to infertility in this vascular disease. Mature spermatozoa are transcriptionally and translationally inert, hence differentially expression of proteins in mature spermatozoa can reflect defects occurring during the spermatogenesis, and help investigating the molecular pathways leading to male infertility (24). A study analyzing the molecular proteome in infertile patients with high SDF compared with fertile donors reported that most DEPs were involved in metabolic and cellular processes, with a catalytic or binding function (25). However, the molecular mechanisms related to DNA integrity, damage, and repair in varicocele patients remain unclear. Therefore, this study aimed to reanalyze the sperm proteomics data (extracted from our previous experiments) in varicocele patients to determine the role of DNA repair molecular mechanisms in varicocele-related infertility by combining a proteomics *in silico* approach with wet-laboratory techniques.

## 2 Materials and Methods

### 2.1 Ethical Statement

This study was performed by analyzing proteomics data collected by the American Center of Reproductive Medicine (Cleveland, Ohio, USA) from previously published studies (26–32). The further recruitment of human subjects for western blotting validation was approved by the Cleveland Clinic Institutional Review Board (IRB#17-422).

### 2.2 Sample and Data Collection

Proteomics data were obtained by analyzing the sperm proteome of varicocele patients (n=50, age: 36.3 ± 7.7 years) attending the Andrology Center (Cleveland Clinic, Cleveland, Ohio, USA) for infertility evaluation between March 2012 and March 2014, and using healthy fertile donors (n=10, age: 40.0 ± 9.8 years) as a control group (26–32). Patients were diagnosed as having unilateral (n=33) or bilateral (n=17) varicocele by genital examination and scrotal palpation, according to the Dubin and Amelar classification (33). A total of 31/33 and 2/33 patients had left or right varicocele, respectively, with 28/33 and 5/33 demonstrating varicocele severity equal to grade 1-2 or 3, respectively. A total of 13/17 and 4/17 bilateral varicocele patients showed varicocele severity to grade 1-2 or 3, respectively. The control group included fertile men who had fathered naturally in the previous two years, with no history of varicocele. The exclusion criteria for both groups were smoking, body mass index (BMI) > 25, presence of female factor infertility, exposure to radiation or chemicals, fever in the previous 90 days, genetic defects, leukocytospermia, infection or inflammation of the reproductive tract, sexually transmitted disease, azoospermia, and moderate oligozoospermia (<10 million sperm/mL).

#### 2.2.1 Semen Analysis, Sperm DNA Fragmentation, and Concentration of Intracellular Reactive Oxygen Species (ROS)

After 3–5 days of sexual abstinence, semen samples were collected by masturbation into a sterile container. After incubation at 37°C for 30 minutes to allow liquefaction, semen parameters (sperm concentration, total motility, and normal morphology) were assessed according to the WHO guidelines (34) within 1 h from the collection. Normal morphology was analyzed according to the strict criteria using Diff-Quik kit (Baxter Healthcare Corporation, Inc., McGaw Park, IL) (35), while leukocyte count was assessed using the peroxidase test (36). SDF was analyzed using terminal deoxynucleotidyl transferase–mediated fluorescein end labeling (TUNEL) assay (Apo-Direct kit, Pharmingen, San Diego, CA) and the flow cytometer FacScan (Becton Dickinson, San Jose, CA) (37). Then, samples were centrifuged at 13,000g for 20 minutes, seminal plasma was removed and sperm samples were stored at − 20°C. Intracellular concentration of reactive oxygen species (ROS) was measured by chemiluminescence assay using luminol (5-amino-2, 3-dihydro-1, 4-phthalazinedione -10 μL, 5 mM). Chemiluminescence was measured for 15 min using a Berthold luminometer (Autolumat Plus 953, Oakridge, TN), and results were expressed as relative light units (RLU)/sec/× 10^6^ sperm (38).

#### 2.2.2 Protein Extraction

Protein extraction was conducted as described previously (26). Briefly, samples from varicoceles and controls were pooled in groups of 5 samples each, based on the minimum sperm concentration needed to perform proteomic analysis. After washing thrice with PBS, samples were incubated in radio-immunoprecipitation assay (RIPA) lysis buffer (Sigma-Aldrich, St. Louis, MO) with proteinase inhibitor cocktail (Roche, Indianapolis, IN) overnight at 4°C. Then, samples were centrifuged at 13,000g for 20 minutes, and the supernatant collected. Protein concentration was analyzed using bicinchoninic acid (BCA) kit (Thermo, Rockford, IL).

#### 2.2.3 Global Proteomics Analysis and Protein Identification

As described previously (26), global proteomic analysis was performed in triplicate and quantified using the label-free spectral counting method. Each sample (15μg) was boiled, and separated by SDS-PAGE (12.5% Tris–HCl) gel at 150 V for 35 minutes. The gel was fixed for 30 minutes in 50% ethanol/10% acetic acid, washed with H_2_O and stained with Coomassie blue. Then, the gel was cut into smaller pieces, which were washed with H_2_O and dehydrated in acetonitrile. They were then reduced with Dithiothreitol (DTT) and alkylated with iodoacetamide, before digesting the gel with trypsin (5 μL 10ng/μL in 50 mM ammonium bicarbonate). After overnight incubation at room temperature, 2 aliquots of 30 μL 50% acetonitrile with 5% formic acid were extracted. The extracts were then resuspended in 1% acetic acid (final volume: ~30 μL) for liquid chromotography mass spectrometer (LC-MS) analysis.

The LC-MS system was a Finnigan LTQ-Orbitrap Elite hybrid mass spectrometer system, while the HPLC column was a Dionex 15 cm × 75 μm internal diameter Acclaim Pepmap C18, 2 μm, 100 Å reversed phase capillary chromatography column. A total of 5μL of each extract was injected, and the peptides eluted by an acetonitrile/0.1% formic acid gradient at a flow rate of 0.25 μL/min were introduced into the source of the mass spectrometer. The microelectrospray ion source was operated at 2.0 kV. The digest was analyzed using the data dependent multi- task capability of the instrument acquiring full scan mass spectra to determine peptide molecular weights and tandem mass spectra (MS/MS) for the amino acid sequence in successive instrument scans.

Proteome Discoverer (version 1.4.1.288) was used to extract tandem mass spectra. Samples were analyzed using Mascot (Matrix Science, London, UK; version 2.3.02), Sequest (Thermo Fisher Scientific, San Jose, CA, USA; version 1.4.0.288) and X! Tandem (The GPM, thegpm.org; version CYCLONE (2010.12.01.1), set to search the human reference with database (33292 entries) assuming trypsin as digestion enzyme (fragment ion mass tolerance: 1.0 Da; parent ion tolerance: 10 parts per million). Scaffold (version Scaffold 4.0.6.1, Proteome Software Inc., Portland, OR) was used to validate protein identification (>95.0% probability by the Peptide Prophet algorithm (39) with Scaffold delta-mass correction; identifications accepted if >99.0% probability to achieve a false detection rate (FDR) <1.0% and at least 2 identified peptides) (40). The comparison of spectral counts normalized using the NSAF (normalized spectral abundance factor) approach was used to identify DEPs (41, 42).

### 2.3 Bioinformatics Analysis

**Figure 1:**
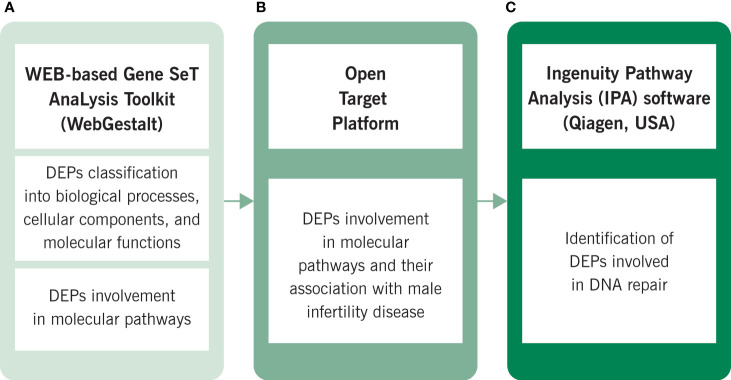
Bioinformatic analysis conducted by using **(A)** WEB-based Gene SeT AnaLysis Toolkit (WebGestalt), **(B)** Open Target Platform and **(C)** Ingenuity Pathway Analysis (IPA). KEGG, Kyoto Encyclopedia of Genes and Genomes.

Proteomics data obtained from spermatozoa of varicocele patients and fertile donors were analyzed using bioinformatics tools. In particular, we further analyzed the DEPs that were previously identified by comparing protein expression in varicocele patients with healthy fertile donors. **Figure 1** summarizes the bioinformatics analysis conducted in this study. Free-online bioinformatics tools such as WEB-based Gene SeT AnaLysis Toolkit (WebGestalt, http://www.webgestalt.org/) (43), and universal protein resource (UniProt, https://www.uniprot.org/) (44) were used to classify DEPs based on the annotation of genes/gene products with gene ontology (GO) terms, focusing on three aspects (biological processes, cellular components, and molecular functions), as well as to identify the pathways involved based on the Kyoto Encyclopedia of Genes and Genomes (KEGG) database. The Open Target Platform (https://www.targetvalidation.org/batch-search) further analyzed the association of DEPs with male infertility conditions. Ingenuity Pathway Analysis (IPA) (Qiagen, USA) was used to identify the DEPs involved in DNA replication, recombination, and repair.

### 2.4 Sperm Protein Extraction, Quantification, and Western Blotting (WB)

Validation through Western Blotting (WB) was conducted in semen samples from a different set of varicocele patients (n=13, 33.5 ± 4.1 years) and fertile control (n=5, 37.0 ± 6.8 years) samples than those analyzed for proteomics, using the same inclusion and exclusion criteria. First, the samples were washed twice with PBS and centrifuged at 4000x g for 7 min to remove the seminal plasma. They were incubated overnight with RIPA buffer (Sigma-Aldrich, St. Louis, MO, USA) (~100 µl/10^6^ sperm) supplemented with Protease Inhibitor Cocktail (cOmpleteTM ULTRA tablets, EDTA-free – Sigma Aldrich, St. Louis, MO, USA) at 4°C to allow the lysis of the sample. The next day, samples were centrifuged at 10,000 × g for 30 min at 4°C, and the supernatant was collected. The concentration of proteins was estimated using the bicinchoninic acid assay (BCA assay, Thermo Fisher Scientific, Waltham, MA, USA).

Based on the bioinformatic analysis, we selected five proteins that were predicted to be differentially expressed in varicocele samples and involved in DNA damage and DNA repair mechanisms: myeloperoxidase (MPO), fatty acid synthase (FASN), mitochondrial aconitate hydratase (ACO2), nucleoporin 93 (NUP93), and 26S proteasome non-ATPase regulatory subunit 14 (PSMD14). Proteins (20 µg) were separated on 4%-15% SDS polyacrylamide gels (Mini-Protean TGX gels, Biorad Inc., Hercules, CA, USA) at constant voltage (90 V) for 2h, and then transferred to polyvinylidene difluoride (PVDF) membrane (GE Healthcare, Marlborough, MA, USA) using the Trans-Blot Cell system (Biorad), for 30 min at 18 V. Membranes were blocked for 1h with 5% milk in Tris-buffered saline with 0.1% Tween (TBST), then washed with TBST (X4, 10 min/each), and incubated overnight at 4°C with the following primary antibodies: αPSMD14 (1:5,000, ab109123, Abcam, Cambridge, UK), αNUP93 (1:1,000, ab53750, Abcam), αACO2 (1:13,000, ab129069, Abcam), αFASN (1:8,000, ab128870, Abcam), αMPO (1:10,000, AF3667-SP, R&D Systems, Minneapolis, MN). The next day, membranes were washed with TBST (X4, 10 min/each), and incubated with the following horseradish peroxidase-conjugated secondary antibodies for 1h at room temperature: donkey α-goat (1:10,000, sc 2020, Santa Cruz Biotechnology, Dallas, TX), goat α-mouse (1:10,000, sc2005, Santa Cruz Biotechnology), goat α-rabbit (1:10,000, ab97200, Abcam). After washing with TBST, the membranes were treated with enhanced chemiluminescence substrate (GE Healthcare) for 1 min to detect proteins. Finally, results were acquired with the ChemiDoc™ MP Imaging System (Bio-Rad), and the density of each band was normalized to the relative total protein lane density, obtained by staining the membranes with colloidal gold total protein stain (Bio-Rad). Densitometric analysis of the results was carried out using the Image Lab™ software (Bio-Rad). Results are expressed as fold change relative to the controls and change in percentage (change%).

### 2.5 Statistical Analysis

Analyses were performed with SPSS, version 25 (32). The Wilcoxon rank sum test was used to compare results from semen analysis, SDF and ROS tests, with significance considered at p <0.05. The 2-tailed Student t-test was applied to compare WB intensity readings.

## 3 Results

### 3.1 Semen Analysis, Sperm DNA Fragmentation, and Concentration of Intracellular Reactive Oxygen Species (ROS)

Samples were analyzed for sperm parameters, the percentage of SDF, and the intracellular ROS concentration as marker of oxidative stress. Results, previously published (26–32), are summarized in **Supplementary Table 1**.

### 3.2 Bioinformatic Analysis Results

**Figure 2:**
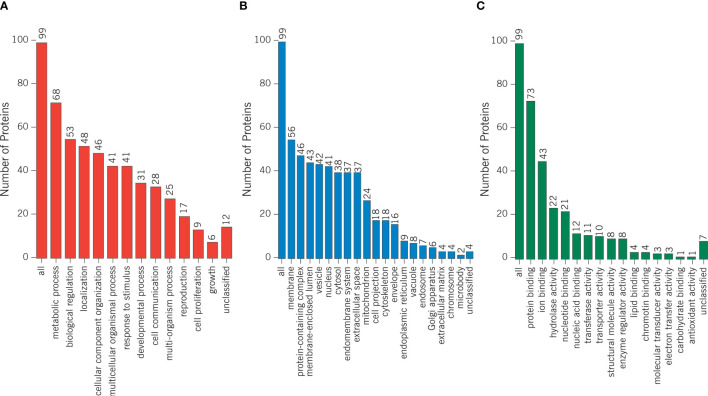
Differentially expressed proteins were categorized based on the functional annotation with GO terms in **(A)** biological processes, **(B)** cellular components, and **(C)** molecular functions.

Proteomics results from varicocele and control groups were already published in previous studies as supplementary data (27, 28). A total of 99 DEPs were identified between the sperm proteome of varicocele and fertile men, with 14 overexpressed and 85 under-expressed proteins (**Supplementary Table 2**). Their involvement in biological processes, cellular components, and molecular function categories was obtained based on the functional annotation of genes/gene products with GO terms using WebGestalt software. The most represented biological process categories were metabolic processes and biological regulation, with 68 and 53 DEPs, respectively, while 56 and 46 DEPs were predictably localized in the membrane or in protein-containing complexes, respectively (**Figure 2**). Based on the GO term categorization for molecular function, they mainly showed protein binding (n=73) or ion binding (n=43) activities (**Figure 2**). Analysis conducted by the open target platform showed the association of several proteins with infertility conditions. Specifically, 66 and 47 of them were reportedly associated with male reproductive system disease and male infertility, respectively (**Table 1**). IPA analysis was conducted to identify the diseases and functions associated with the DEP list. Out of the various functions identified by the software, we focused our attention on the category “DNA replication, recombination, and repair”. In particular, the analysis showed 2 (FASN, MPO) and 3 (ACO2, NUP93, and PSMD14) proteins, which were significantly over- and under-expressed, respectively (P <0.03) in varicocele samples and reported to be involved in DNA repair function. These proteins were chosen for *in vitro* validation by WB.

**Table 1:** List of differentially expressed proteins (DEPs) that are associated with male infertility, as reported by Open Targets Platform analysis.

Disease full name	Relevance (p-value)	**Number of DEPs**	DEPs
Male reproductive system disease	4.00E-22	66	RSPH9, TTC25, PRKAR1A, FN1, PRKACA, IQGAP1, NDRG1, NDUFS1, CUL3, AK7, B2M, ACSL6, PDHA2, PSMD2, HIBADH, ATP1A4, H1-7, NME5, CCDC42, ODF2, CACNA2D2, RSPH6A, ITGB2, IDH3A, TEKT3, HSPA4L, HSPA2, CLGN, CS, ABHD10, IDH3B, ACADS, HADHA, FLNB, HMOX2, GAA, RUVBL1, DYNLL2, ACAT1, TGM4, SPA17, MPO, PSMD13, ACLY, EIF3F, HNRNPM, APOA1, DPCD, PSMD14, LETM1, PGAM5, TTR, EIF3I, KARS1, CPD, RAN, ACO2, CFAP58, AAAS, NUP93, IMMT, RABAC1, AZU1, CAPZA1, GFPT1, ITGAM
Male infertility	8.00E-18	47	RSPH9, TTC25, FN1, IQGAP1, NDUFS1, AK7, PDHA2, NME5, H1-7, CCDC42, ATP1A4, RSPH6A, CACNA2D2, ODF2, PRKACA, ITGB2, IDH3A, TEKT3, HSPA4L, HSPA2, CLGN, CS, ABHD10, IDH3B, PRKAR1A, NDRG1, ACADS, HADHA, FLNB, GAA, HMOX2, B2M, MPO, DPCD, APOA1, PGAM5, TTR, CUL3, KARS1, ACO2, RAN, CFAP58, AAAS, ACSL6, SPA17, TGM4, ACLY
Male infertility due to sperm motility disorder	9.00E-13	23	RSPH9, TTC25, AK7, CACNA2D2, NME5, ATP1A4, H1-7, RSPH6A, PRKACA, ODF2, TEKT3, CCDC42, PRKAR1A, HSPA4L, IDH3B, ABHD10, CLGN, HSPA2, DPCD, APOA1, MPO, ACO2, RAN
Male infertility due to sperm disorder	5.00E-11	24	RSPH9, TTC25, AK7, PDHA2, NME5, H1-7, ATP1A4, RSPH6A, CCDC42, CACNA2D2, PRKACA, ODF2, TEKT3, HSPA4L, PRKAR1A, ABHD10, HSPA2, IDH3B, CLGN, DPCD, APOA1, MPO, ACO2, RAN
Male infertility due to gonadal dysgenesis or sperm disorder	3.00E-10	24	RSPH9, TTC25, AK7, PDHA2, NME5, H1-7, ATP1A4, RSPH6A, CCDC42, CACNA2D2, PRKACA, ODF2, TEKT3, HSPA4L, PRKAR1A, ABHD10, HSPA2, CLGN, IDH3B, DPCD, APOA1, MPO, RAN, ACO2
Male infertility due to obstructive azoospermia	9.00E-09	19	NDUFS1, AK7, CCDC42, ODF2, PRKACA, ATP1A4, NME5, ABHD10, RSPH6A, HSPA2, H1-7, CLGN, IDH3B, FN1, B2M, KARS1, PRKAR1A, MPO, APOA1
Male infertility with teratozoospermia due to single gene mutation	1.00E-08	14	PRKACA, H1-7, ATP1A4, CCDC42, ODF2, AK7, RSPH6A, ABHD10, CLGN, HSPA2, IDH3B, TEKT3, HSPA4L, NME5
Non-syndromic male infertility due to sperm motility disorder	1.00E-08	11	AK7, ATP1A4, H1-7, RSPH6A, CCDC42, ODF2, TEKT3, NME5, PRKACA, PRKAR1A, HSPA4L
Male infertility with spermatogenesis disorder due to single gene mutation	5.00E-08	16	AK7, PDHA2, CCDC42, H1-7, ATP1A4, PRKACA, NME5, ODF2, RSPH6A, HSPA4L, CLGN, ABHD10, IDH3B, HSPA2, TEKT3, PRKAR1A
Male infertility with spermatogenesis disorder	5.00E-08	16	AK7, PDHA2, CCDC42, H1-7, ATP1A4, PRKACA, NME5, ODF2, RSPH6A, HSPA4L, ABHD10, CLGN, HSPA2, IDH3B, TEKT3, PRKAR1A
Male infertility due to obstructive azoospermia of genetic origin	7.00E-08	16	NDUFS1, AK7, CCDC42, ATP1A4, PRKACA, ODF2, NME5, ABHD10, CLGN, RSPH6A, H1-7, IDH3B, HSPA2, FN1, B2M, PRKAR1A
Male infertility with azoospermia or oligozoospermia due to single gene mutation	0.00005	10	AK7, PDHA2, CCDC42, NME5, H1-7, ATP1A4, RSPH6A, HSPA4L, ODF2, TEKT3

### 3.3 Validation by Western Blotting

**Figure 3:**
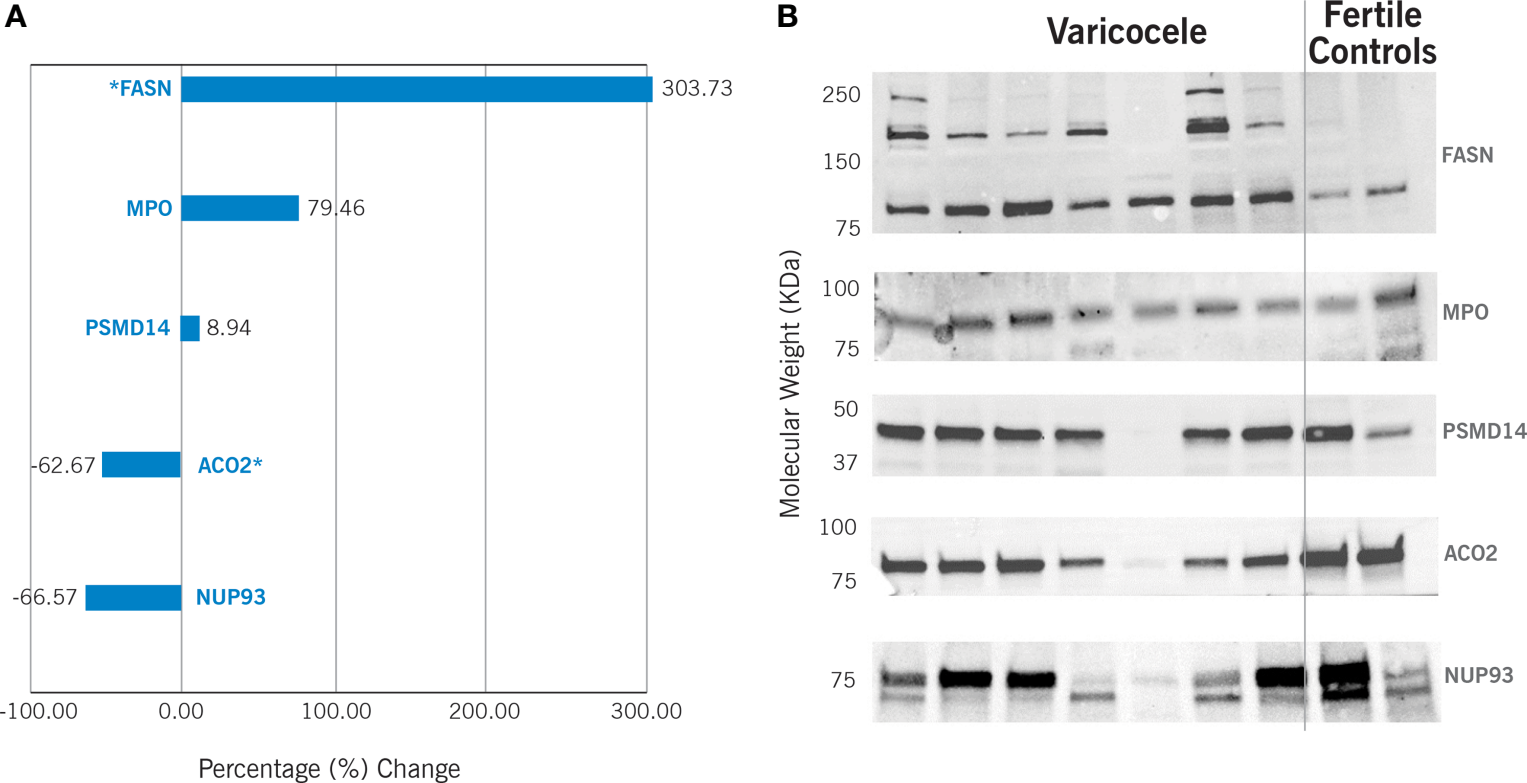
Western Blotting results in varicocele samples are expressed as percentage (%) change in comparison to fertile controls. This is calculated by analyzing 13 varicocele samples and 5 controls. **(A)** Graphic representation of the differential protein expression. *P<0.05. **(B)** Example of Western blotting results. FASN - fatty acid synthase (predicted molecular weight – pMW: 273kDa); MPO – myeloperoxidase (pMW: 83kDa); PSMD14 - 26S proteasome non-ATPase regulatory subunit 14 (pMW: 34.6 kDa); ACO2 - mitochondrial aconitate hydratase (pMW: 85.4 kDa); NUP93 - nucleoporin 93 (pMW: 93.5 kDa).

The differential expression of the proteins ACO2, NUP93, FASN, MPO, and PSMD14 was validated by WB (**Figure 3**). Our results confirmed significant downregulation of ACO2 protein (P = 0.0001) in varicocele samples, with a fold change equal to 0.37 and change% of -62.7%. Similarly, NUP93 was also under-expressed (fold change: 0.33, change%: -66.6%), although this was not statistically significant (P=0.38). WB confirmed the overexpression of FASN (fold change: 4.04, change%: 303.7%, P=0.014) and MPO (fold change: 1.79, change%: 79.5%), although the latter was not statistically significant (P=0.14). However, WB did not show any variation in PSMD14 expression (fold change: 1.09, change%: 8.9%, P=0.78).

## 4 Discussion

Sperm DNA integrity plays a significant role in determining male fertility potential and achieving pregnancy. On one hand, our study confirmed previous reports of poor semen quality, and higher SDF and oxidative stress in varicocele patients (4, 8). On the other hand, by re-analyzing our previously published proteomics data (26–32), we identified proteins involved in DNA repair mechanisms in varicocele sperm samples. To our knowledge, this is the first study using an *in silico* approach (based on bioinformatics tools) that identifies proteins involved in DNA repair mechanisms in patients diagnosed with varicocele.

**Figure 4:**
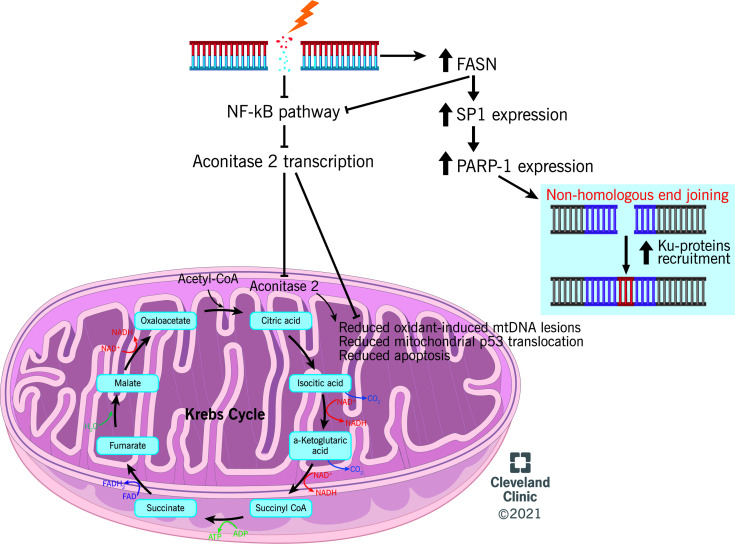
Proposed mechanism explaining the association between differential proteins expression (aconitase 2 and FASN) and DNA damage and repair in varicocele condition.

We identified 99 proteins that were differentially expressed in varicocele; of these, several had been previously described in the literature to have a role in DNA repair mechanisms and have been associated with male infertility. In particular, we confirmed by WB analysis the altered expression of ACO2 and FASN, which was previously observed by mass spectrometry, and proposed a model linking their differential expression and DNA damage/repair mechanisms in varicocele (**Figure 4**).

ACO2 is an enzyme of the tricarboxylic acid (TCA) cycle, which catalyzes the isomerization of citrate to isocitrate *via* cis-aconitate (45). The TCA cycle is particularly important for sperm motility because it generates adenosine triphosphate (ATP). An association between reduced ACO2 expression and asthenozoospermia has been previously reported (46). ACO2 is also involved in the prevention of oxidant-induced mitochondrial dysfunction in alveolar epithelial cells, p53 translocation to the mitochondria, and intrinsic apoptosis (47). In case of DNA damage, signaling pathways that control cell proliferation are regulated to inhibit the progression of the cell cycle (48). NF-κB is responsible for ACO2 transcription (49). Based on our preliminary observations, an association between ACO2 expression and the regulation of proliferative cellular pathways might be speculated (**Figure 4**); however, further studies must be conducted before considering the reduced expression of ACO2 as an indirect marker of NF-κB under-regulation and, consequently, DNA damage.

FASN is a multifunctional enzyme that catalyzes the *de novo* biosynthesis of long-chain saturated fatty acids. Specifically, it catalyzes the synthesis of palmitate from acetyl-CoA and malonyl-CoA during the anabolic metabolism of fatty acids (50). This protein has seven catalytic activities and a site for the binding of the prosthetic group 4’-phosphopantetheine of the acyl carrier protein (ACP) domain (51). Overexpression of FASN was confirmed by WB analysis in semen samples from couples with unexplained recurrent pregnancy loss (52). In somatic cells, homologous recombination (HR) is the main molecular mechanism for DSB repair, which occurs by using the sister chromatid as a template. However, spermatids are haploid cells, and they do not have a chromatin sister; hence, sperm rely on the NHEJ pathway for DSB repair (53). It has been reported that FASN can suppress NF-κB pathway and induce the overexpression of specificity protein 1 (SP1), which in turn increases the expression of poly(ADP-ribose) polymerase 1 (PARP-1) and facilitates the recruitment of Ku proteins (54). On one side, the inhibition of NF-κB pathway mediated by FASN would be consistent with the mechanism proposed for ACO2 regulation. On the opposite side, FASN overexpression may suggest an enhanced recruitment of mediators of the NHEJ pathway, which is an alternative cellular mechanism for DSB repair (55) (**Figure 4**). Hence, FASN overexpression in varicocele sperm samples may represent a cellular effort to counterbalance DSB induced by varicocele disease through the activation of the NHEJ pathway.

*In silico* analysis identified two additional proteins (NUP93 and MPO) that were predicted to be significantly altered in varicocele sperm. However, validation by WB did not show statistical significance when confirming the altered trend.

NUPs are the main component of the nuclear pore complex (NPC) in eukaryotic cells. This massive structure extends across the nuclear envelope, forming a gateway that regulates the flow of macromolecules between the nucleus and cytoplasm (56). It is a target of caspase cysteine proteases in apoptosis and localizes both to the basket of the pore and to the nuclear entry of the central gated channel of the pore with NUP98, NUP205, and NUP153 (56, 57). In human sperm, NUP93 was previously reported to be significantly underexpressed in the sperm proteome of patients with testicular cancer seminoma, although the authors did not provide any explanation for such alterations (58). Another NUP factor, NUP153, is specifically required for 53BP1 nuclear import, a mediator of cellular DNA damage response, and a tumor suppressor whose accumulation on damaged chromatin promotes DNA repair and enhances DNA repair response (59). Disorder in NUP153 stops 53BP1 from entering the nuclei of newly formed daughter cells. Hence, reduced NUP153 expression results in increased DNA damage (59). Considering the role of NUP153 in DNA repair response and its binding with other NUPs as NPC components, we can speculate that NUP93 might be involved in this type of cellular regulation, explaining why our *in silico* analysis predicted downregulation of NUP93 in varicocele patients.

MPO is part of the host defense system of polymorphonuclear leukocytes. This enzyme catalyzes the production of hypohalous acids, primarily hypochlorous acid, which is responsible for microbicidal activity against a wide range of organisms (60). Neutrophils are powerful inhibitors of the NER pathway. In particular, high MPO immunoreactivity was associated with a major reduction in NER capacity (61, 62). In the liver, high MPO expression was associated with reduced DNA damage recognition and NER capacity, with an effect on hepatic inflammation (61, 62). In seminal plasma, high levels of MPO are correlated with low semen quality (63). In our study, the predicted overexpression of MPO may be related to reduced NER activity and increased varicocele-induced inflammation.

Results from SDF and ROS testing confirm the role of oxidative stress in varicocele condition. We speculated the involvement of identified DEPs in pathways related to DNA damage/repair in varicocele samples. However, the proteins identified in our study may be differentially expressed in poor semen quality samples, irrespective of the presence of varicocele. In this regard, varicocele condition might increase the percentage of poor quality sperm that harbor these changes. The lack of a second control group including abnormal semen samples without varicocele is a limitation of this study. In addition, the fertility status of patients with varicocele is unknown. The comparison of proteomics data from both fertile and infertile varicocele patients might be of interest to decipher the alterations in molecular mechanisms that influence fertility potential. Moreover, WB validation did not confirm a statistical alteration in NUP93, MPO, and PSMD14 proteins. This may be due to the small sample size analyzed, as well as a different spectrum of sensitivity of both techniques for protein detection.

In conclusion, our *in silico* study describes the molecular mechanisms related to DNA repair that may be altered in varicocele conditions. We identified ACO2 and FASN as possible mediators of DNA repair, whose expression was altered in varicocele. Larger studies are needed to confirm these preliminary results and investigate protein function in *in vitro* and *in vivo* models.

## Data Availability Statement

The datasets presented in this study can be found in online repositories. The names of the repository/repositories and accession number(s) can be found in the article/**Supplementary Material**.

## Ethics Statement

The studies involving human participants were reviewed and approved by Cleveland Clinic IRB. The patients/participants provided their written informed consent to participate in this study.

## Author Contributions

Conception and design: RF and PP. Collection of data: RF and SD. Data analysis and interpretation: RF, SD, and PP. Manuscript writing: All authors. All authors contributed to the article and approved the submitted version.

## Funding

This study was supported by the American Center for Reproductive Medicine, Cleveland Clinic, Cleveland, Ohio, USA.

## Conflict of Interest

Author RH was employed by company LogixX Pharma.

The remaining authors declare that the research was conducted in the absence of any commercial or financial relationships that could be construed as a potential conflict of interest.

## Publisher’s Note

All claims expressed in this article are solely those of the authors and do not necessarily represent those of their affiliated organizations, or those of the publisher, the editors and the reviewers. Any product that may be evaluated in this article, or claim that may be made by its manufacturer, is not guaranteed or endorsed by the publisher.
