# Supplementary material for: In Silico Sperm Proteome Analysis to Investigate DNA Repair Mechanisms in Varicocele Patients
Source: Front Endocrinol (Lausanne). 2021 Dec 17;12:757592. doi: 10.3389/fendo.2021.757592 (PMC8719329; doi:10.3389/fendo.2021.757592)
Supplement: Supplementary file 1 [file Table_1.docx]

**Supplementary Table 1.** Results from semen analysis, sperm DNA fragmentation (SDF) and intracellular reactive oxygen species (ROS) testing are reported for both varicoceles and controls.

| **Semen Parameters** | **Fertile healthy men (n=10)** | **Varicocele patients**  **(n=50)** | **P value** |
| --- | --- | --- | --- |
| Sperm concentration (10^6^/mL) | 69.90 ± 37.65 | 29.49 ± 33.22 | < 0.002 |
| Sperm motility (%) | 57.1 ± 16.0 | 41.3 ± 18.4 | < 0.023 |
| Normal sperm morphology (%) | 8.4 ± 3.7 | 2.4 ± 1.9 | < 0.001 |
| ROS levels (RLU/sec/10^6^ sperm) | 142.7 (36.2, 337.7) | 896.1 (165.6, 2990.5) | < 0.008 |
| Sperm DNA fragmentation (%) | 8.5 to 18.2 | 3.3 to 48.3 | < 0.009 |

Sperm concentration, motility and morphology values are presented as mean ± SD. ROS levels are mentioned as median (25th, 75th percentile), whereas sperm DNA fragmentation are represented as lower and upper limit. For all values, P < 0.05 indicate a significant difference based on the Mann-Whitney test. RLU: relative light units.

**Supplementary Table 2.** Differentially expressed proteins (DEPs) with the normalized spectral abundance factor (NSAF) ratio.

| **SN** | **Underexpressed Proteins** | **NSAF ratio** |
| --- | --- | --- |
|  | Uncharacterized protein c22orf43 | 0.0 |
|  | Nucleoporin p58/p45 isoform a | 0.0 |
|  | Uncharacterized protein c9orf135 | 0.0 |
|  | Coiled-coil domain-containing protein 42A isoform 1 | 0.0 |
|  | HD domain-containing protein 2 | 0.0 |
|  | Protein DPCD | 0.0 |
|  | V-type proton atpase subunit B, brain isoform | 0.0 |
|  | Voltage-dependent calcium channel subunit alpha-2/delta-2 isoform c (CACNA2D2) | 0.0 |
|  | V-type proton atpase subunit E 1 isoform a | 0.1 |
|  | Leucine-rich repeat-containing protein 23 isoform a | 0.1 |
|  | Heterogeneous nuclear ribonucleoprotein M isoform a | 0.1 |
|  | Syntaxin-12 | 0.0 |
|  | Tetratricopeptide repeat protein 25 | 0.0 |
|  | Apolipoprotein A-I preproprotein (APOA1) | 0.0 |
|  | Nuclear pore glycoprotein p62 | 0.0 |
|  | Serine/threonine-protein phosphatase PGAM5, mitochondrial isoform 1 (PGAM5) | 0.0 |
|  | Methionyl-trna synthetase, cytoplasmic | 0.1 |
|  | Testis-specific H1 histone | 0.1 |
|  | UPF0733 protein c2orf88 | 0.1 |
|  | Cullin-3 | 0.1 |
|  | Camp-dependent protein kinase catalytic subunit alpha isoform 2 | 0.1 |
|  | GTP-binding nuclear protein Ran (RAN) | 0.1 |
|  | 2-oxoglutarate dehydrogenase, mitochondrial isoform 3 precursor | 0.1 |
|  | Transthyretin precursor | 0.1 |
|  | Carboxypeptidase D isoform 1 precursor (CPD) | 0.1 |
|  | Long-chain-fatty-acid--coa ligase 6 isoform e | 0.1 |
|  | UBX domain-containing protein 11 isoform 1 | 0.1 |
|  | Eukaryotic translation initiation factor 3 subunit I | 0.2 |
|  | Abhydrolase domain-containing protein 10, mitochondrial precursor | 0.2 |
|  | Adenylate kinase 7 | 0.2 |
|  | Mitochondrial import receptor subunit TOM22 homolog | 0.2 |
|  | 26S proteasome non-atpase regulatory subunit 2 | 0.2 |
|  | F-actin-capping protein subunit alpha-1 | 0.2 |
|  | Delta(3,5)-Delta(2,4)-dienoyl-coa isomerase, mitochondrial precursor | 0.2 |
|  | EF-hand domain-containing protein 1 isoform 1 | 0.2 |
|  | 26S proteasome non-atpase regulatory subunit 14 | 0.2 |
|  | Prenylated Rab acceptor protein 1 | 0.2 |
|  | Melanoma inhibitory activity protein 3 precursor (MIA3) | 0.2 |
|  | Enoyl-coa hydratase, mitochondrial | 0.2 |
|  | Eukaryotic translation initiation factor 3 subunit F | 0.2 |
|  | Pro-cathepsin H preproprotein | 0.2 |
|  | Lysyl-trna synthetase isoform 1 | 0.2 |
|  | LETM1 and EF-hand domain-containing protein 1, mitochondrial precursor | 0.2 |
|  | Isocitrate dehydrogenase [NAD] subunit beta, mitochondrial isoform a precursor | 0.3 |
|  | Calmegin precursor | 0.3 |
|  | Coiled-coil domain-containing protein 147 | 0.3 |
|  | Lysosomal alpha-glucosidase preproprotein | 0.1 |
|  | Protein disulfide-isomerase A4 precursor (PDIA4) | 0.1 |
|  | Nucleoporin p54 | 0.2 |
|  | NADH-ubiquinone oxidoreductase 75 subunit, mitochondrial isoform 1 (NDUFS1) | 0.2 |
|  | T-complex protein 1 subunit zeta-2 isoform 1 | 0.3 |
|  | Uncharacterized protein c9orf9 | 0.3 |
|  | Heme oxygenase 2 (HMOX2) | 0.3 |
|  | Nucleoside diphosphate kinase homolog 5 (NME5) | 0.3 |
|  | Sperm surface protein Sp17 | 0.3 |
|  | Camp-dependent protein kinase type I-alpha regulatory subunit (PRKAR1A) | 0.3 |
|  | Radial spoke head protein 6 homolog A | 0.4 |
|  | Mitochondrial inner membrane protein isoform 3 (IMMT) | 0.4 |
|  | Citrate synthase, mitochondrial precursor (ACLY) | 0.4 |
|  | Pyruvate dehydrogenase E1 component subunit alpha, testis-specific form, mitochondrial precursor | 0.4 |
|  | Nuclear pore complex protein Nup93 isoform 1 | 0.4 |
|  | Beta-2-microglobulin precursor (B2M) | 0.4 |
|  | Elongation factor 1-delta isoform 1 | 0.4 |
|  | Short-chain specific acyl-coa dehydrogenase, mitochondrial precursor | 0.4 |
|  | Valyl-trna ligase (VARS1) | 0.4 |
|  | Tripeptidyl-peptidase 2 | 0.4 |
|  | 26S proteasome non-atpase regulatory subunit 13 isoform 1 | 0.4 |
|  | Isochorismatase domain-containing protein 2, mitochondrial isoform 2 | 0.4 |
|  | Radial spoke head protein 9 homolog isoform 1 | 0.4 |
|  | Sodium/potassium-transporting atpase subunit alpha-4 isoform 1 | 0.4 |
|  | Isocitrate dehydrogenase [NAD] subunit alpha, mitochondrial precursor | 0.4 |
|  | Heat shock 70 protein 4L | 0.4 |
|  | Cytochrome c oxidase subunit 5B, mitochondrial precursor | 0.4 |
|  | Dynein light chain 2, cytoplasmic | 0.4 |
|  | Pyruvate dehydrogenase E1 component subunit beta, mitochondrial isoform 1 precursor | 0.4 |
|  | Leucine-rich repeat-containing protein 37A precursor | 0.4 |
|  | 3-hydroxyisobutyrate dehydrogenase, mitochondrial precursor | 0.5 |
|  | Acetyl-coa acetyltransferase, mitochondrial precursor (ACAT1) | 0.5 |
|  | Aconitate hydratase, mitochondrial precursor (ACO2) | 0.4 |
|  | Acrosin-binding protein precursor | 0.4 |
|  | Leucine-rich repeat-containing protein 37B precursor | 0.4 |
|  | Ruvb-like 1 (RUVBL1) | 0.5 |
|  | Trifunctional enzyme subunit alpha, mitochondrial precursor | 0.6 |
|  | Cytochrome b-c1 complex subunit 2, mitochondrial precursor (UQCRC2) | 0.6 |
|  | Heat shock-related 70 protein 2 | 0.6 |
| **SN** | **Overexpressed Proteins** | **NSAF ratio** |
|  | Tektin-3 | 1.7 |
|  | Outer dense fiber protein 2 isoform 3 | 1.6 |
|  | Fibronectin isoform 3 preproprotein (FN1) | 1.9 |
|  | Ras gtpase-activating-like protein IQGAP1 (IQGAP1) | 2.0 |
|  | Cytosolic non-specific dipeptidase isoform 1 (CNDP2) | 2.1 |
|  | Glucosamine--fructose-6-phosphate aminotransferase [isomerizing] 1 isoform 1 | 2.2 |
|  | Protein NDRG1 (NDRG1) | 2.6 |
|  | ATP-citrate synthase isoform 1 | 2.8 |
|  | Protein-glutamine gamma-glutamyltransferase 4 | 2.9 |
|  | Filamin-B isoform 2 (FLNB) | 3.7 |
|  | Myeloperoxidase precursor (MPO) | 4.5 |
|  | Azurocidin preproprotein | 17.3 |
|  | Integrin alpha-M isoform 1 precursor (ITGAM) | 25.7 |
|  | Integrin beta-2 precursor | 5.6 |
